# Development of a Job Retention Vocational Rehabilitation Intervention for People with Multiple Sclerosis Following the Person-Based Approach

**DOI:** 10.1177/02692155241235956

**Published:** 2024-02-28

**Authors:** Blanca De Dios Pérez, Roshan das Nair, Kathryn Radford

**Affiliations:** 1 Centre for Rehabilitation and Ageing Research, School of Medicine, 6123 University of Nottingham, Nottingham, UK; 2 NIHR Nottingham Biomedical Research Centre, Nottingham, UK; 3 Mental Health & Clinical Neurosciences, School of Medicine, 6123 University of Nottingham, Nottingham, UK; 4 Institute of Mental Health, Nottinghamshire NHS Foundation Trust, Nottingham, UK; 5 Health Research, 555969 SINTEF Digital, Trondheim, Norway

**Keywords:** Vocational rehabilitation, multiple sclerosis, job retention, intervention development, person-Based approach, complex intervention

## Abstract

**Objective:**

To describe the process of developing a job retention vocational rehabilitation intervention for people with multiple sclerosis.

**Design:**

We used the person-based approach, to develop interventions through an iterative process incorporating stakeholders’ views, resulting in an intervention that is likely to be more acceptable, contextually relevant, and implementable for end-users. Phase 1 combined the results of a systematic review and interview study to develop the guiding principles and intervention logic model. Phase 2 involved conceptual testing and refining the intervention with stakeholder feedback. We present the final intervention following the template for intervention description and replication.

**Participants:**

We recruited 20 participants for Phase 1 (10 people with multiple sclerosis, four employers, six healthcare professionals), and 10 stakeholders (three people with multiple sclerosis, seven healthcare professionals) for Phase 2 to contribute to the intervention refinement process.

**Results:**

Stakeholders described the need for an individually tailored intervention to support people with multiple sclerosis to manage symptoms and workplace relationships. A stepped-care approach and remote support were deemed essential. The resulting intervention involves an initial assessment of employment needs, vocational goal setting, up to 10 h of tailored support (e.g., reasonable adjustments, employer engagement, legal rights), and a final review to discuss future steps. People with multiple sclerosis can include their employer for advice to optimise the management of the employee with multiple sclerosis at work.

**Conclusion:**

The person-based approach provided a rigorous framework to systematically understand the vocational needs of people with multiple sclerosis and develop a vocational rehabilitation intervention.

## Introduction

Multiple Sclerosis is the most common chronic neurological condition affecting young adults and the most common non-traumatic disability in adults.^1,2^ It affects over one million people in Europe.^3^ People are diagnosed with multiple sclerosis in their prime working years; unfortunately, many leave the workplace prematurely or become unemployed soon after diagnosis.^4^ The unemployment rate for people with multiple sclerosis is estimated at 80%.^5^

Vocational Rehabilitation interventions aim to support those with illness or disability to remain or return to employment or other useful occupation.^6^ These are complex interventions that include multiple interacting components, have a variety of outcomes, and require a high degree of flexibility/tailoring to the needs of the person.^7,8^

Evidence for the effectiveness of vocational rehabilitation for people with multiple sclerosis, is inconclusive, in part due to the limited available evidence.^9^ Few vocational rehabilitation interventions have been developed, implemented, and evaluated. Common components of existing interventions include support with symptom management,^10^ neuropsychological support,^11^ or support developing job seeking skills,^12^ depending on the aim of the intervention. However, while these components have been used, it is unclear whether they are effective at supporting people with multiple sclerosis at work. Thus, there is a need to develop vocational rehabilitation interventions to support people with multiple sclerosis to remain at work and gather evidence about the effectiveness of common intervention components used in these interventions.

The updated Medical Research Council Framework for developing, evaluating, and implementing complex interventions^13,14^ provides a guide to developing complex interventions taking into consideration the available evidence and combining this with further primary research to gain a deeper understanding of the issue being explored.^13^ This framework signposts relevant methods that aid in intervention development.

One such method is the person-based approach.^15^ This method supports the intervention development process by obtaining in-depth knowledge about the views and needs of those who will use and/or deliver the intervention through mixed-methods research, to enhance future acceptability and feasibility.^15^ This method was selected because of the target population-centre approach,^16^ driving the need to include potential end users early in the intervention development process to develop a more contextually relevant intervention.

This paper illustrates the process of developing a job retention vocational rehabilitation intervention to support people with multiple sclerosis to remain at work following the person-based approach. This process is reported in accordance with the guideline for reporting for intervention development studies Guided Checklist (supplementary material 1).^17^

## Methods

Following the person-based approach, the intervention development was split into two phases. Phase 1 combined the results of a systematic review and interview study to develop guiding principles and an intervention logic model. Phase 2 involved conceptual testing and refining the preliminary intervention with stakeholder feedback.

In Phase 1, the intervention *design objectives* were selected according to the needs identified in the qualitative study on the experiences of people with multiple sclerosis at work.^18^ The intervention *features* were selected based on the findings from a systematic review (supplementary material 2), and preferences reported by the participants involved in the qualitative study.^18,19^

An intervention logic model was developed at this stage to illustrate the structure and process of the intervention and how the intervention components would integrate and interact with each other.^14,20^ The main sections of the logic model include the resources, activities, underlying mechanisms, and outcomes. The logic model's “activities” section was the first to be populated. The activities refer to the intervention components delivered as part of the identified interventions in the systematic review (supplementary material 2), and proposed support needs identified in the qualitative study. These activities were then matched with the resources needed to conduct them. The ‘outcomes’ section was completed by identifying which outcomes the intervention might influence based on the activities to address problems reported by people with multiple sclerosis at work. Once the activities, resources, and outcomes sections were complete, we identified the underlying mechanisms that bring about change.^21^ These were identified by discussing the assumptions about how the intervention as a whole and individual activities work(s) to influence an effect on the outcome (e.g., how can the intervention support the person with multiple sclerosis to remain at work?). Following the development of the Phase 1 logic model, the data were reviewed, and reorganised to avoid repetition and to create a logical path to reflect how different sections of the logic model fit together.

The primary researcher (BDP) recorded areas where there was insufficient information or the links between the different logic model sections were unclear to address them with stakeholders in Phase 2.

Phase 2 involved collecting stakeholder feedback to refine the guiding principles and intervention logic model. Stakeholders were identified through a network of patient and public involvement representatives (from the Nottingham Multiple Sclerosis Research Group's patient and public involvement network), advertising through social media, local voluntary groups (e.g., Multiple Sclerosis Society), and previous research participants. We included two groups of stakeholders: people with multiple sclerosis for their lived experience and as future ‘end users’ of the intervention, and healthcare professionals for their knowledge of healthcare systems and processes affecting the implementation of any new intervention and for their expertise in supporting people with illness or disability at work. Stakeholders with multiple sclerosis were included in the study if they had a confirmed diagnosis of multiple sclerosis and were in paid employment. The inclusion criterion for healthcare professionals was having previous experience of delivering vocational rehabilitation or supporting people with multiple sclerosis to return or remain at work.

We completed the United Kingdome Health Research Authority self-assessment to identify the need for ethical approval. The assessment confirmed that there was no need for National Health Service approval, as the study was considered a stakeholder consultation. No personal data were collected apart from publicly available information (e.g., name, job title of the healthcare professional group), the issues discussed were not sensitive or confidential, and there was no risk for potential disclosure or reporting obligations. Therefore, no formal ethical approval was required to collect the stakeholder’ feedback.

All stakeholders were informed about the study's purpose and their right to withdraw. Stakeholders consented to participate and needed to be able to communicate in English.

Stakeholders were given a set of slides describing the intervention, its guiding principles and the logic model before their feedback session. The session aimed to guide the stakeholders through the proposed intervention process to seek their views and identify gaps. Through an iterative process, their ideas were incorporated into the intervention. Because of the complexity of the logic model, the primary researcher first described each section of the logic model to facilitate its understanding. The following aspects were addressed:

- Intervention components (e.g., identifying reasonable adjustments)
- Resources (e.g., fatigue management guidelines, booklets about funding schemes)
- Delivery mode and intensity of the intervention (e.g., how many hours, where)
- Contextual factors (e.g., healthcare professionals involved, support available)
- Underlying mechanisms (e.g., trust, shared understanding)
- Relevant outcomes (e.g., job retention)

The discussion was conducted individually in-person or via telephone according to the preference of the stakeholder, following a conversational style guided by the logic model, and lasted between 40 to 60 min. The primary researcher (BDP) took notes of the points raised, and ideas discussed and recommended changes to the intervention.

A patient and public involvement representative with multiple sclerosis was involved in the development and review of research documents for Phases 1 and 2.

To ensure that the intervention is described comprehensively, we present it following the Template for Intervention Description and Replication Checklist.^22^

## Results

### Intervention Development Phase 1

The primary objective of the intervention was to support people with multiple sclerosis to remain at work for as long as they wish. We recruited 20 participants (10 people with multiple sclerosis, four employers, and six healthcare professionals) for the interview study conducted as part of Phase 1.^18^ These interviews provided insight into the target behaviours, challenges, and needs the intervention should address. The design objectives and key intervention features to address the target challenges were developed based on evidence from the systematic review. The target issues identified that the intervention should address were:

1. Interaction between multiple sclerosis symptoms, environment and workplace characteristics can create barriers to job retention.^23^ Research has shown that people with higher “work instability” (the mismatch between the capacity of an individual and the job requirements)^24^ are at risk of losing their jobs.^25,26^ All research participant groups identified this target need.
2. Generally, people with multiple sclerosis and their employers lack awareness about multiple sclerosis, legal rights and how to manage their condition in the workplace. It is common for people with multiple sclerosis not to seek support at work until the problems are too difficult to manage.^27,28^ Furthermore, colleagues and employers do not fully understand the array of symptoms, especially those that are not visible (e.g., fatigue).^29^ Healthcare professionals and participants with multiple sclerosis identified the need for the intervention components focussed on providing education to people with multiple sclerosis and employers about symptoms, their impact on work, and legal rights.
3. Vocational rehabilitation interventions for people with multiple sclerosis are characterised by high drop-out rates.^30^ Common reasons for dropping out are a high level of disability, a high workload and stress at work.^30^ Healthcare professionals and participants with multiple sclerosis identified this target challenge for the intervention.

During the qualitative interview study, there was general consensus of ideas around how best to support people with multiple sclerosis at work. However, some ideas were unique to certain participant groups. For example, participants with multiple sclerosis generally felt unsupported at work because of co-workers and employer attitudes. Employers were more focused on providing physical workplace accommodations than understanding multiple sclerosis or providing emotional support. This is a clear mismatch between the employee's needs and the available support. Healthcare professionals highlighted that people with multiple sclerosis only tend to seek help with employment once their MS was affecting their workability and they were trying to compensate for their performance at work by working additional hours. Not seeking timely support when MS symptoms start to affect the person at work, can lead to poor performance and affect work relationships. These views need to be considered during the intervention development process to target the key behaviours that could pose challenges at work. Considering this information, we developed the guiding principles presented in Table 1.

**Table 1.** Guiding principles phase 1.

Target issue	Design objective	Key intervention features	Supporting evidence
The experiences of people with multiple sclerosis at work differ according to their multiple sclerosis and work context.	To improve the workability of employed people with multiple sclerosis	Provide support tailored to the needs of each person. Provide support soon after diagnosis. Prioritise relevant/urgent issues.	Vocational rehabilitation interventions for people with long-term neurological conditions and by extension multiple sclerosis need to be individually tailored due to the variability in employment and disease-related factors causing difficulties at work.^44^ Early intervention is recommended for people with chronic illnesses and can be understood as providing support soon after diagnosis or before a crisis arises.^27,28,30,45–47^ Urgent issues should be prioritised in the intervention to reduce work instability.^44^
People with multiple sclerosis are not always aware of the support they need at work or their employment rights.	To empower people with multiple sclerosis at work	Inform and advise people with multiple sclerosis about disclosure and legal rights. Educate people with multiple sclerosis about strategies to self-manage multiple sclerosis symptoms (e.g., fatigue). Educate people with multiple sclerosis, employers, and colleagues about multiple sclerosis.	Educating the person with multiple sclerosis about their legal rights and symptoms will empower them to address future problems at work.^28,38^ Symptom management is a common intervention component to help the person manage their condition at work.^11,48^ Employer engagement has been recommended for these interventions and can lead to improved workplace relationships.^10,46^
Lack of time as a result of high workload, family responsibilities, and the impact of multiple sclerosis symptoms can hamper participation in the intervention.	To facilitate participation in the intervention	Flexible support including *face-to-face* support plus *telephone* and/or *email* contact. Appointments are booked according to the person's availability. Re-accessible support.	Telerehabilitation is an acceptable method to support people with multiple sclerosis with employment.^12,49^ People with multiple sclerosis should be able to access the vocational rehabilitation intervention as required without having to request additional referrals to the intervention.^44^

**Figure 1.**
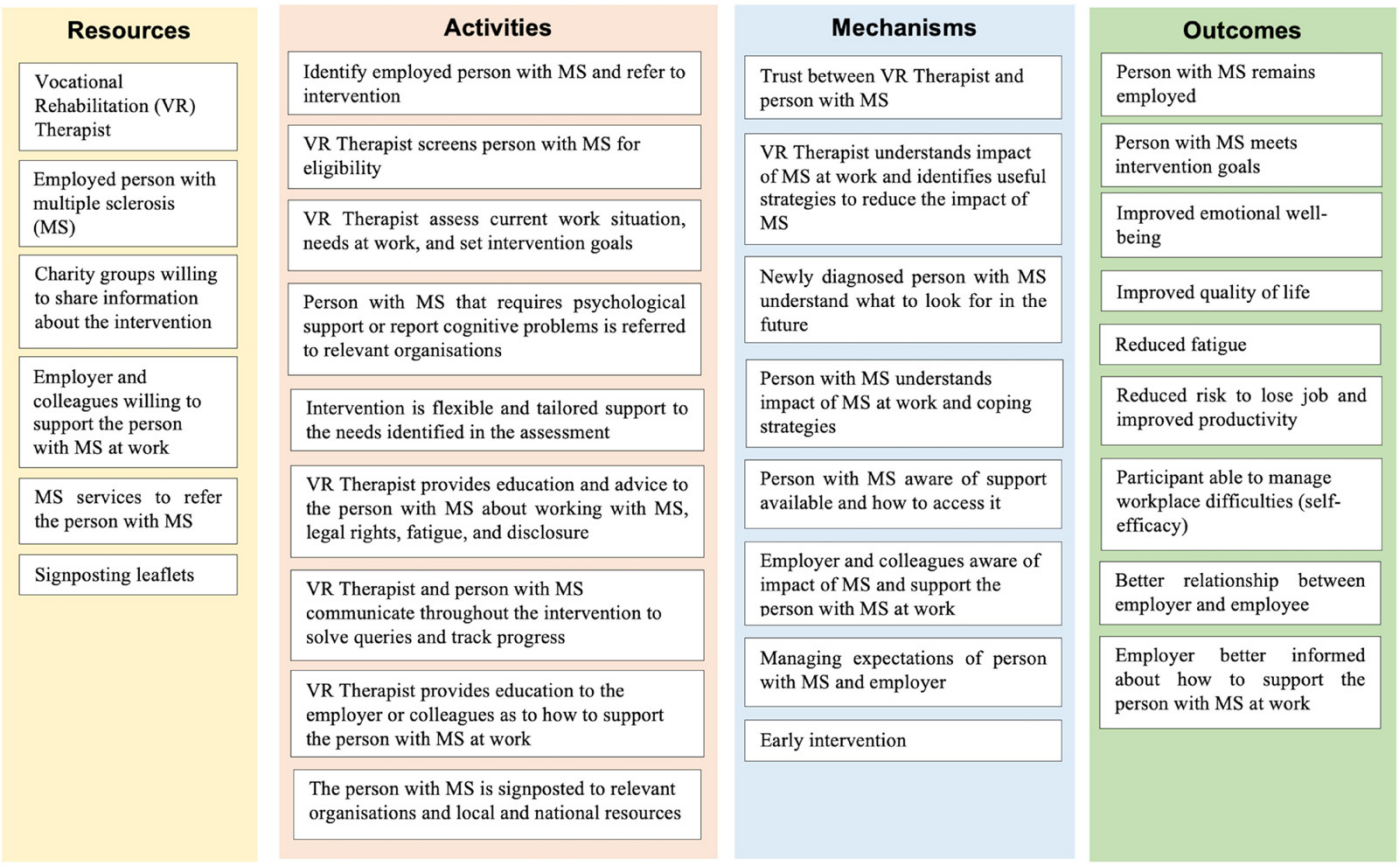
Logic model phase 1.

The development of the Phase 1 logic model (Figure 1) started by populating the four sections of the logic model (resources, activities, mechanisms, and outcomes) using an iterative process based on the data gathered through the systematic review and interview study.

Key intervention activities identified in Phase 1 refer to the need to assess the impact of multiple sclerosis at work, education and advice about legal rights, and symptom management. The systematic review did not identify the resources needed to deliver the intervention; thus, the interviews focused on understanding the necessary resources, barriers and facilitators to deliver this type of support. At this point, it was clear that further stakeholder feedback was required to illustrate how the intervention would work in practice.

### Intervention Development Phase 2

We recruited ten stakeholders (eight women, and two men) for this study, including three people with multiple sclerosis and seven healthcare professionals (six Occupational Therapists and one Neurologist).

The stakeholders noted the relevance of relationships with co-workers (including the employer) on the job performance and satisfaction of the person with multiple sclerosis. This issue was previously included in the design objectives, but the stakeholders explained that it should be separate. Therefore, the guiding principles were refined to incorporate this change.

The stakeholders identified an additional target behaviour, challenges or needs for the intervention. This referred to the need to improve awareness about the support available to remain at work and the legal responsibility of the employer to provide reasonable accommodation for people with multiple sclerosis under the Equality Act 2010.^31^

Stakeholders discussed that employers commonly request support from occupational health departments (where available), but these professionals did not always understand the nuances of multiple sclerosis. This may lead to people with multiple sclerosis receiving generic advice to manage their condition at work from them. Therefore, the stakeholders explained that the intervention should include a component to identify and request reasonable adjustments. The Phase 2 guiding principles are presented in Table 2.

**Table 2.** Guiding principles phase 2.

Target issue	Design objective	Key intervention features	Supporting evidence
The experiences of people with MS at work differ according to their multiple sclerosis and work context.	To improve the workability of employed people with multiple sclerosis	Provide support tailored to the needs of each person. Provide support soon after diagnosis. Prioritise relevant/urgent issues.	**Evidence from literature:** Vocational rehabilitation for people with multiple sclerosis needs to be individually tailored due to the variability in employment and disease-related factors causing difficulties at work.^44^ Early intervention is recommended for people with chronic illnesses and can be understood as providing support soon after diagnosis or before a crisis arises.^27,28,30,45–47^ Urgent issues should be prioritised in the intervention to reduce work instability.^44^ **Stakeholder ID:** OT_01; OT_02; OT_04; MS_03
People with multiple sclerosis are not always aware of the support they need at work or their legal rights at work.	To empower people with multiple sclerosis at work and increase awareness about legal rights	Inform and advise people with multiple sclerosis about disclosure and legal rights. Educate people with multiple sclerosis about strategies to self-manage symptoms.	**Evidence from literature:** Educating the person with multiple sclerosis about their legal rights and symptoms will empower them to address future problems at work.^28,38^ Symptom management is a common intervention component to help the person manage their condition at work.^11,48^ **Stakeholder ID:** Neuro_01; OT_04
Co-workers are not sympathetic with people with MS because they do not understand the impact of multiple sclerosis on their colleague	To increase the awareness of multiple sclerosis and its symptoms for colleagues and employers.	Educate employers, and colleagues about multiple sclerosis	Employer engagement has been recommended for these interventions and can lead to improved workplace relationships.^10,46^ **Stakeholder ID:** MS_01-03; OT_01; OT_04; OT_06
Not all companies have an occupational health department to recommend support at work, and employers do not always provide the support.	To identify reasonable adjustments for the person with multiple sclerosis	Assessment of needs at work. Support the employer and person with multiple sclerosis by identifying reasonable adjustments.	**Evidence from literature:** Conducting a detailed assessment of employment needs can identify barriers to job retention and support to reduce the barriers.^11,44,48^ People with multiple sclerosis are protected under the Equality Act 2010, and their employers are obliged to provide them with reasonable adjustments.^31^ **Stakeholder ID:** OT_01; OT_02; Neuro_01
Lack of time as a result of high workload, family responsibilities, and the impact of multiple sclerosis symptoms can hamper people with multiple sclerosis’ participation in the intervention.	To facilitate the participation in the intervention	Flexible support including *face-to-face* support plus *telephone* and/or *email* contact. Appointments are booked according to the person's availability. Re-accessible support.	**Evidence from literature:** Telerehabilitation is an acceptable method to support people with multiple sclerosis with employment.^12,49^ People with multiple sclerosis should be able to access the vocational rehabilitation intervention as required without having to request additional referrals to the intervention.^44^ **Stakeholder ID:** MS_01; MS_03; Neuro_01; OT_03; OT_06

**Figure 2.**
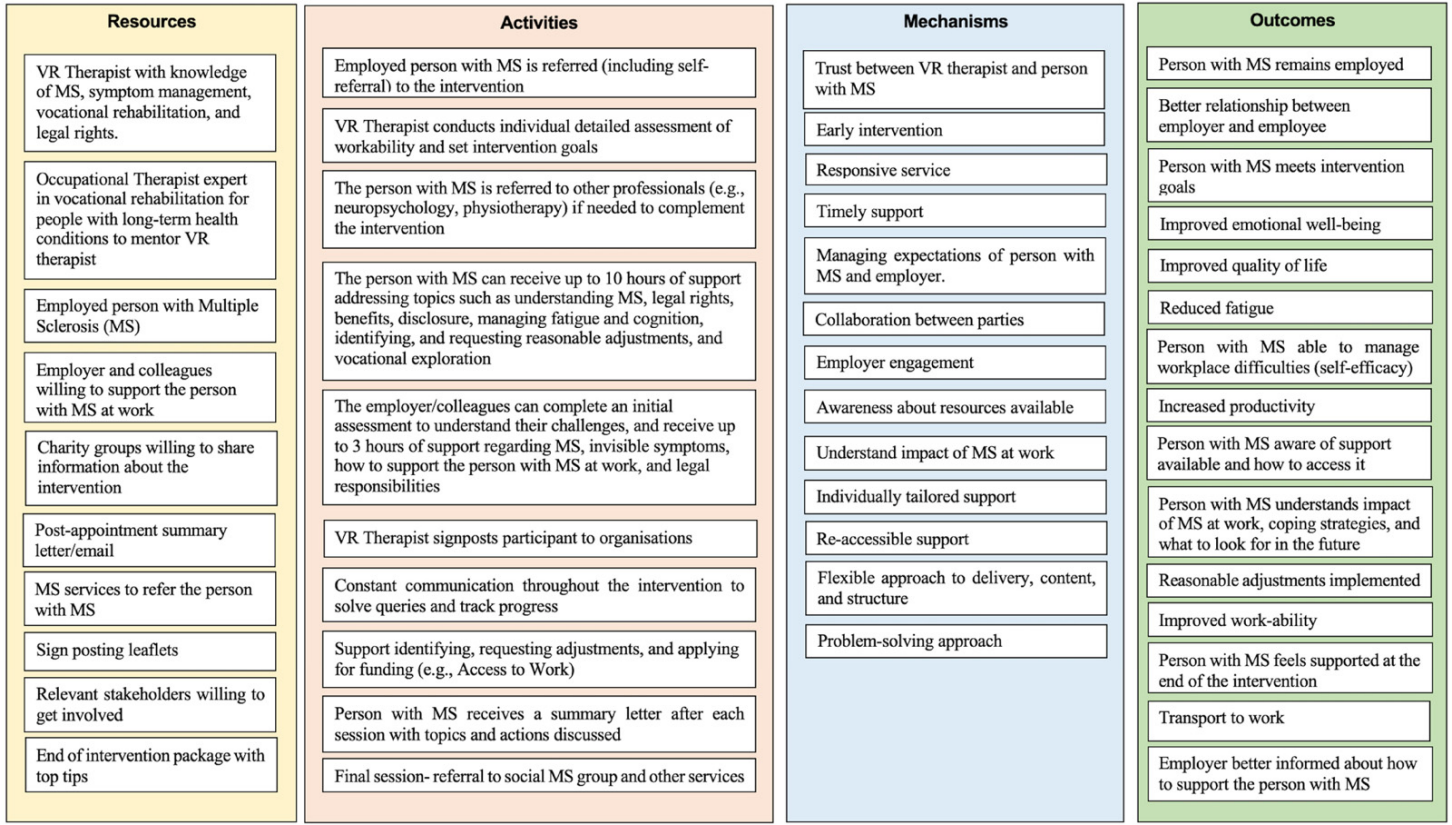
Logic model phase 2.

Informed by the guiding principles, the intervention logic model was refined with stakeholders and changed substantially from Phase 1 to Phase 2 (Figure 2).

The resources were extended by mapping the activities to the resources and identifying resource gaps from the initial logic model. The stakeholders suggested arranging the activities temporally to reflect the intervention process from recruitment to discharge.

Stakeholders believed that the vocational rehabilitation professional (person delivering the intervention) should be an Occupational Therapist with experience delivering vocational rehabilitation for people with multiple sclerosis and other complex health conditions. Stakeholders also believed that Psychologists with experience working with people with multiple sclerosis, knowledge of disability discrimination law, the ability to identify compensatory strategies to manage multiple sclerosis symptoms at work, and an in-depth understanding of multiple sclerosis could be suited for this role. The Psychologist could address common employment issues, address milder mental health issues, share information, and signpost the person with multiple sclerosis (and employer) to local and national resources.

Stakeholders expressed interest in individual support as opposed to group sessions because employment issues are specific to the person with multiple sclerosis and their workplace characteristics. The idea of providing an “end-of-intervention package” emerged at this stage. This involves providing top tips and a summary of the intervention in the final appointment.

Stakeholders believed 10 h of support would be sufficient to address the most common employment concerns; but those with ongoing needs (e.g., psychological issues, employment tribunal hearings) might need to be referred to specialist support such as legal advisors or union representatives.

Regarding the intervention length, the vocational rehabilitation interventions identified in the systematic review ranged between four weeks and 12 months. The stakeholders believed that a three-month intervention with varying hours of support (between 1–10 h) following a stepped-care model would be beneficial for most employed people with multiple sclerosis. Thus, those who are not experiencing problems at work could receive fewer hours. Stakeholders noted that certain complex issues, such as support with an employment tribunal hearing, would require more than three months. Additionally, because of the progressive nature of multiple sclerosis, stakeholders suggested that the intervention should include easy referral pathways, such as self-referrals or ‘open cases’, as needs may change in the future.

Regarding the outcomes and mechanisms, new ideas were discussed that led to a clearer differentiation between individual and measurable outcomes, as well as mechanisms (i.e., collaborative approach) that were underrepresented in the preliminary logic model. This was further refined by reviewing the underlying mechanisms of vocational rehabilitation interventions for people with other long-term neurological conditions. Mechanisms such as collaborative approach, early intervention, and tailored were seen as essential.

### Job Retention VR Intervention

The resulting intervention developed through Phases 1 and 2 involves a three-month job retention intervention including an initial interview, vocational goal setting, and up to 10 h of individually tailored support dependent on the needs and/or goals of the person with multiple sclerosis. The intervention content is selected from a menu of components (i.e., activities presented in logic model) addressing common problems at work, and a final appointment to discuss the progress made and future steps.

The intervention can be delivered by an occupational therapist and/or psychologist according to the needs of the person with multiple sclerosis at work.

The professional delivering the intervention should adopt the role of case coordinator, referring or signposting the person with multiple sclerosis to relevant organisations (e.g., Government funds, local charities), supporting them with the referral process, and adopting an educational role, providing information and support with applications and identifying reasonable adjustments. Where delivered collaboratively, the Occupational Therapist and Psychologist should collaborate to address complex barriers to job retention, such as phased return to work, discrimination at work, assessment of function concerning workability, long-term sick leave, and employment tribunals, amongst others.

Once a person with multiple sclerosis has agreed to be involved in the intervention, the first appointment involves completing the initial interview (lasting approximately one hour). The aim is to understand the following aspects:

- Demographic and professional information.
- Clinical characteristics (e.g., years with the condition, symptoms, impact of symptoms).
- Work characteristics: Information regarding the job duties, and support received from the employer.
- Discussing what is important for the person with MS at work.
- Detailed assessment of the needs of the person at work.

The initial interview is followed by vocational goal setting, with the support of the vocational rehabilitation professional. The goals should be SMART (specific, measurable, achievable, realistic/relevant, and timed) and evaluated at the end of the intervention.^32,33^ The content of the sessions should be selected to achieve the intervention goals.

The initial appointment will structure the whole intervention, thus, if the person with multiple sclerosis feels fatigued, it can be split in two. At this appointment, the person with multiple sclerosis should be asked if they are interested in involving their employer in the intervention.

The intervention for people with multiple sclerosis can involve between one and 10 h of individually-tailored support on topics relevant to them. For example, education about multiple sclerosis, legal rights, support with disclosure, fatigue management, managing cognitive problems at work, advice about reasonable adjustments, employer engagement, signposting to local and national resources, managing emotions (e.g., stress and anxiety), long-term career planning, and referrals to other services. The support can be divided into sessions that typically last one hour; the sessions can be shorter if needed.

Each appointment after the initial interview starts by reviewing the progress made to date and addressing new queries. After each session, the person with multiple sclerosis receives a letter by post or via email describing the topics discussed in the session and further educational resources (if needed).

We estimated that not everybody would need 10 h of support because this will vary according to their situation at work. Stakeholders suggested that the intervention should be flexible in the content and delivery mode, and re-accessible so that people can opt to receive information during the first session and decide later if they would like to receive further support.

The employers (if/when included) will have the opportunity to complete an initial interview to explore their knowledge and experience supporting the employee with multiple sclerosis at work, and up to three hours of support on topics such as reasonable adjustments, legal responsibilities, or understanding multiple sclerosis. The amount of support was selected for practical reasons, to ensure employers could incorporate the intervention within their busy work schedules.

The final appointment of the intervention was designed to help the vocational rehabilitation professional and person with multiple sclerosis reflect on the progress made and content learnt. The person with multiple sclerosis could receive a document summarising further resources and top tips via email or post. The document could also include information about peer support groups. At the end of the intervention, those with further needs should be referred to the organisation or professional that can address the problem, for example, the services of neuropsychologists, legal advisors, and physiotherapists. The resulting intervention following the template for intervention description and replication checklist can be found in supplementary material 3.

## Discussion

This manuscript presents the steps taken to develop a job retention vocational rehabilitation intervention to support people with multiple sclerosis at work following the person-based approach. The person-based approach enabled us to develop an intervention through an iterative process that requires quick analysis and integration of data to optimise the process of intervention development.

We identified five guiding principles to target issues identified in the systematic review and interviews study. There was a need to improve the experiences of people with multiple sclerosis at work from a biopsychosocial perspective^34^ to understand how multiple sclerosis symptoms interact with the environment hampering job retention. For this reason, the proposed intervention adopts a holistic approach targeting both disease-related and environmental factors to overcome barriers.

Stakeholders also reported the relevance of identifying reasonable adjustments at work to identify barriers to job retention. The International Classification of Functioning, Disability and Health^35^ is useful for understanding the complex relationship between multiple sclerosis and work, as it recognises that disability is created or removed according to the interaction between environmental and disease-related factors. The International Classification of Functioning, Disability and Health has been used as a conceptual framework to understand vocational rehabilitation because of its comprehensive account of the needs of the person, health condition and environment.^36,37^

The intervention also strives to empower the person with multiple sclerosis by providing them with education about their condition, and support for self-management of symptoms. Previous research has shown that education about multiple sclerosis is beneficial to overcome future issues that may appear as a result of the progressive character of the condition.^28,38^

Issues regarding the awareness of the support available and co-workers’ attitudes were also identified in this study. These aim to support the person with multiple sclerosis, employer, and co-workers to understand better the impact multiple sclerosis has at work. Employer engagement is a common component of vocational rehabilitation interventions because it can help to improve workplace relationships which are essential to job retention.^10,39^

Finally, it was clear that the intervention needed to be flexible in terms of delivery mode, content and frequency of sessions to facilitate its delivery alongside the everyday activities of the person with MS and the employer. Vocational rehabilitation interventions are characterised by high dropout levels.^30^ Therefore, key features include remote appointments (e.g., videoconference, telephone call), flexible and out-of-office hours appointment options, and shorter sessions.

It is increasingly important to identify the underlying mechanisms of complex interventions as an approach to understanding how interventions lead to different outcomes.^14^ The identification of underlying mechanisms in vocational rehabilitation interventions is of utmost importance because vocational rehabilitation is a highly individualised intervention, of great complexity, delivered to people who experience diverse symptoms and workplace difficulties.^21,40^

We identified several mechanisms in this study (e.g., early intervention, open access) that have been previously described in the vocational rehabilitation literature for people with long-term neurological conditions.^41^ However, we recognise that even after refining the intervention logic model, some outcomes could be considered mechanisms and vice versa. To understand how vocational rehabilitation works, we need to explore the responses that certain intervention components trigger in participants to help us achieve our desired outcome (job retention).^21^

This outcome can only be achieved when the circumstances allow optimal interaction between context, the individual's psychological adjustment, and stakeholder collaboration.^5,37^

One of the main strengths of this study was following a novel approach to developing a vocational rehabilitation intervention through an iterative process of combining literature, interviews, and stakeholder feedback. Combining evidence-based knowledge with feedback from potential end-users and service providers helped us understand the complex interaction between health and work.

A weakness of this study is that we mostly included Occupational Therapists as stakeholders in the healthcare professionals’ group. This was likely caused because of their unique training to deal with employment. Another limitation of the study (Phase 2) was that it included only three people with multiple sclerosis, and we did not collect their clinical and employment data. Their experiences may have influenced the content and structure of the intervention. In our qualitative study, we focused mostly on understanding the problems that people with multiple sclerosis experience at work and interviewed 10 people with multiple sclerosis. We used the findings from that study to inform the intervention development process (as explained in the methods). Therefore, after having a thorough understanding of the problems, our approach shifted towards understanding the type of support that could help address these issues.

The intervention developed as part of this manuscript was tested as a mixed-methods single case series with people with multiple sclerosis and their employers (where included), and it was associated with improved goal attainment at four time points up to 12 months post-intervention.^42^ Future research should explore the underlying mechanisms that underpin vocational rehabilitation interventions for people with multiple sclerosis. Realist methodologies provide the basis to identify these mechanisms and inform a programme theory about how the intervention will work, under what circumstances, for whom, and why.^43^

In conclusion, the person-based approach provided rigorous guidelines to develop a job retention vocational rehabilitation intervention for people with multiple sclerosis. The resulting intervention addresses a wide range of employment issues that people with multiple sclerosis can experience at work and incorporates characteristics that can increase the acceptability of the intervention.

**Clinical messages**

- Vocational Rehabilitation for people with multiple sclerosis should focus on self-management of symptoms, education about legal rights, and how to explain the impact of multiple sclerosis at work.
- Collaboration between the employer, employee, and co-workers is essential to accommodate the needs of the person with multiple sclerosis at work.

## Supplemental Material

sj-docx-1-cre-10.1177_02692155241235956 - Supplemental material for Development of a Job Retention Vocational Rehabilitation Intervention for People with Multiple Sclerosis Following the Person-Based Approach Supplemental material, sj-docx-1-cre-10.1177_02692155241235956 for Development of a Job Retention Vocational Rehabilitation Intervention for People with Multiple Sclerosis Following the Person-Based Approach by Blanca De Dios Pérez, Roshan das Nair and Kathryn Radford in Clinical Rehabilitation
