# Supplementary material for: Development of a Job Retention Vocational Rehabilitation Intervention for People with Multiple Sclerosis Following the Person-Based Approach
Source: Clin Rehabil. 2024 Feb 28;38(7):965–78. doi: 10.1177/02692155241235956 (PMC11118787; doi:10.1177/02692155241235956)
Supplement: sj-docx-1-cre-10.1177_02692155241235956 - Supplemental material for Development of a Job Retention Vocational Rehabilitation Intervention for People with Multiple Sclerosis Following the Person-Based Approach [file sj-docx-1-cre-10.1177_02692155241235956.docx]

**Supplementary Materials**

Supplementary Material 1: Guided Checklist

| Item Description | Explanation | Page in manuscript | Other* |
| --- | --- | --- | --- |
| 1. Report the context for which the intervention was developed | Understanding the context in which an intervention was developed informs readers about the suitability and transferability of the intervention to the context in which they are considering evaluating, adapting, or using the intervention. The context here can include place, organisational and wider socio-political factors that may influence the development and/or delivery of the intervention. | Page 5-7 |  |
| 1. Report the purpose of the intervention development process | Clearly describing the purpose of the intervention specifies what it sets out to achieve. The purpose may be informed by research priorities, for example those identified in systematic reviews, evidence gaps set out in practice guidance such as The National Institute for Health and Care Excellence or specific prioritisation exercises such as those undertaken with patients and practitioners through the James Lind Alliance. | Pages 5-8 |  |
| 1. Report the target population for the intervention development process. | The target population is the population that will potentially benefit from the intervention – this may include patients, clinicians, and/or members of the public. If the target population is clearly described, then readers will be able to understand the relevance of the intervention to their own research or practice. Health inequalities, gender and ethnicity are features of the target population that may be relevant to intervention development processes. | Page 5 |  |
| 1. Report how any published intervention development approach contributed to the development process | Many formal intervention development approaches exist and are used to guide the intervention development process (e.g., 6Squid or The Person-Based Approach to Intervention Development). Where a formal intervention development approach is used, it is helpful to describe the process that was followed, including any deviations. More general approaches to intervention development also exist and have been categorised as follows: - Target Population-centred intervention development; evidence and theory-based intervention development; partnership intervention development; implementation-based intervention development; efficacy-based intervention development; step or phased-based intervention development; and intervention-specific intervention development. These approaches do not always have specific guidance that describes their use. Nevertheless, it is helpful to give a rich description of how any published approach was operationalised. |  | Supplementary material 2 |
| 1. Report how evidence from different sources informed the intervention development process. | Intervention development is often based on published evidence and/or primary data that has been collected to inform the intervention development process. It is useful to describe and reference all forms of evidence and data that have informed the development of the intervention because evidence bases can change rapidly, and to explain the manner in which the evidence and/or data was used. Understanding what evidence was and was not available at the time of intervention development can help readers to assess transferability to their current situation. | Pages 5-7 | [1,2] |
| 1. Report how/if published theory informed the intervention development process. | Reporting whether and how theory informed the intervention development process aids the reader’s understanding of the theoretical rationale that underpins the intervention. Though not mentioned in the e-Delphi or consensus meeting, it became increasingly apparent through the development of our guidance that this theory item could relate to either existing published theory or programme theory. | Pages 8-11 | Supplementary material 2 |
| 1. Report any use of components from an existing intervention in the current intervention development process. | Some interventions are developed with components that have been adopted from existing interventions. Clearly identifying components that have been adopted or adapted and acknowledging their original source helps the reader to understand and distinguish between the novel and adopted components of the new intervention. | n/a |  |
| 1. Report any guiding principles, people or factors that were prioritised when making decisions during the intervention development process. | Reporting any guiding principles that governed the development of the application helps the reader to understand the authors’ reasoning behind the decisions that were made. These could include the examples of particular populations who views are being considered when designing the intervention, the modality that is viewed as being most appropriate, design features considered important for the target population, or the potential for the intervention to be scaled up. | Tables 1 & 2 |  |
| 1. Report how stakeholders contributed to the intervention development process. | Potential stakeholders can include patient and community representatives, local and national policy makers, health care providers and those paying for or commissioning health care. Each of these groups may influence the intervention development process in different ways. Specifying how differing groups of stakeholders contributed to the intervention development process helps the reader to understand how stakeholders were involved and the degree of influence they had on the overall process. Further detail on how to integrate stakeholder contributions within intervention reporting are available. | Pages 5-8 |  |
| 1. Report how the intervention changed in content and format from the start of the intervention development process. | Intervention development is frequently an iterative process. The conclusion of the initial phase of intervention development does not necessarily mean that all uncertainties have been addressed. It is helpful to list remaining uncertainties such as the intervention intensity, mode of delivery, materials, procedures, or type of location that the intervention is most suitable for. This can guide other researchers to potential future areas of research and practitioners about uncertainties relevant to their healthcare context. | Pages 8-13 |  |
| 1. Report any changes to interventions required or likely to be required for subgroups. | Specifying any changes that the intervention development team perceive are required for the intervention to be delivered or tailored to specific subgroups enables readers to understand the applicability of the intervention to their target population or context. These changes could include changes to personnel delivering the intervention, to the content of the intervention, or to the mode of delivery of the intervention. | Page 11-13 |  |
| 1. Report important uncertainties at the end of the intervention development process. | Intervention development is frequently an iterative process. The conclusion of the initial phase of intervention development does not necessarily mean that all uncertainties have been addressed. It is helpful to list remaining uncertainties such as the intervention intensity, mode of delivery, materials, procedures, or type of location that the intervention is most suitable for. This can guide other researchers to potential future areas of research and practitioners about uncertainties relevant to their healthcare context. | Pages 13-16 |  |
| 1. Follow TIDieR guidance when describing the developed intervention. | Interventions have been poorly reported for a number of years. In response to this, internationally recognized guidance has been published to support the high-quality reporting of health care Interventions and public health interventions. This guidance should therefore be followed when describing a developed intervention. | Pages 11-13 | Supplementary material 3 |
| 1. Report the intervention development process in an open access format. | Unless reports of intervention development are available people considering using an intervention cannot understand the process that was undertaken and make a judgement about its appropriateness to their context. It also limits cumulative learning about intervention development methodology and observed consequences at later evaluation, translation, and implementation stages. Reporting intervention development in an open access (Gold or Green) publishing format increases the accessibility and visibility of intervention development research and makes it more likely to be read and used. Potential platforms for open access publication of intervention development include open access journal publications, freely accessible funder reports or a study webpage that details the intervention development process. | Manuscript submitted for publication. | |
| *e.g., if the item is reported elsewhere, then the location of this information can be stated here. | | | |

1. B. de Dios Pérez, K. Radford, R. das Nair, Experiences of people with multiple sclerosis at work: Towards the understanding of the needs for a job retention vocational rehabilitation intervention, Work. 72 (2022) 303–313.
2. B. de Dios Perez, K. Radford, K. Powers, N. Evangelou, R. das Nair, Vocational rehabilitation for people with multiple sclerosis: a systematic review, PROSPERO. (2019).

**Supplementary Material 2: Summary of Systematic Review Findings**

The following supplementary material presents an overview of the findings and gaps of knowledge from a systematic review of vocational rehabilitation for people with multiple sclerosis. The key findings are presented following the Template for Intervention Description and Replication (TIDieR) Checklist^1^.

The full review is currently being updated to be submitted for publication.

Protocol

The protocol for this systematic review was registered on PROSPERO [CRD42019118526] (<https://www.crd.york.ac.uk/prospero/>).

Overview of results

From the fourteen studies identified reporting thirteen interventions, twelve were research studies, and two were expert opinion articles (as classified by the National Service Framework typology)^2^. Information about five interventions was extracted from the expert opinion articles. Each expert opinion article reported information regarding more than one intervention. The original reports of those interventions were not available even after contacting the primary author and organisation that funded the study.

Table S 1 Overview of systematic review findings and gaps of knowledge.

| Intervention Item | Data extracted | Gap of knowledge |
| --- | --- | --- |
| Intervention Rationale | The main ideas reported were:   - Empowerment through education: Sharing resources and supporting self-management of symptoms^3,4^. - Healthcare providers do not have time or experience to deal with employment issues, thus, there is a need for a professional to support their services^4^. - Services need to be re-accessible to account for the progressive character of multiple sclerosis^5,6^. - Collaborative approach between employer and employee with a disability^5,7^. - Focus on modifying the environment to support the person at work^5,8^. | Limited information regarding how the intervention rationale informed the intervention development and whether/how enablement and behaviour change theories were included to develop the intervention. |
| Materials | - Interview guides to conduct the vocational assessment. - Fatigue management guides. - Neuropsychological assessments. - Resources to signpost participants to national organisations. - Videos and reading materials. | The was missing information regarding the resources used for each intervention component, and resources shared with participants. |
| Procedures/ Intervention Components | The most common intervention components were:   - Vocational assessment: Understand skills and preferences - Vocational counselling: Education about the impact of multiple sclerosis at work, how to request reasonable adjustments, disability discrimination, support with disclosure, and managing expectations. - Workability assessment - Employer engagement - Assessment of work requirements - Case management: Liaison with other professionals - Rehabilitation technology - Goal setting - Symptom management: Support managing cognition and fatigue at work. - Job-seeking skills: Find work placements, further training, write a curriculum vitae, and how to approach employers. - Job follow along   Other components: Neuropsychological assessment, cognitive rehabilitation, psychotherapy, physical/occupational therapy^4,6^. | The intervention components varied according to interventions aiming to support people to remain at work (e.g., vocational counselling, employer engagement), to those who needed support to return to work (e.g., job-seeking skills).  The intervention components aimed to understand the skills, abilities, and preferences of the person, and how they impact work performance.  Limited information regarding the amount of support provided for each intervention component. |
| Who provided | The professionals included in the delivery of the intervention were: Psychologists and employment specialists^4,6^, vocational rehabilitation counsellor^8^, rehabilitation counsellor^7,9-11^, rehabilitation professionals ^3,7^ and occupational therapists^5^. One intervention did not include direct contact with professionals (self-help intervention)^12,13^. | No information regarding the training provided to deliver the intervention, qualifications, or areas of expertise of the professional delivering the intervention. |
| How/ Where | The most common delivery modes were telephone and face-to-face support (n=6) ^4–6,8,10,11^ , group interventions (n=5) ^3,7^ and remotely only (n=2)^12,14^.  Two interventions integrated the support within existing healthcare services ^5,15^ | No clear rationale behind the selection of group, remote, or individual sessions.  No information regarding who benefits the most from each type of support. |
| When and how much | The longest intervention length was 12 months^4,5^. The rest of interventions ranged between 4 and 16 weeks^3,7,8,12,13^.  The literature reported on the need of providing early intervention^16^. | Intervention length varied according to complexity of support provided. Two interventions provided support according to the needs of the client.  From the literature it was not clear what it is considered as “early intervention” or what support would be most beneficial for a person newly diagnosed. |
| Tailoring | Support tailored to the employment, clinical and demographic characteristics of the person with multiple sclerosis^5,6,16^.  Interventions are characterised by providing information to increase awareness of the person with multiple sclerosis and the employer (see intervention content) ^5,12,13,15^.  Employers were only included if the person with multiple sclerosis agreed to it. | The interventions did not provide information regarding the reasoning behind why participants required longer or shorter interventions. |
| Intervention outcomes | Outcomes were available for five interventions ^4– 6,12,13^.  Employment-related outcomes: employment status, the impact of multiple sclerosis at work, and job-seeking activities.  Other outcomes: self-efficacy, quality of life, fatigue, and mood. | The intervention outcomes were not available for all studies. |

**References**

1. Hoffmann T, Glasziou P, Boutron I, Milne R, Perera R, Moher D. Better reporting of interventions: template for intervention description and replication (TIDieR) checklist and guide. Br Med J 2014. https://doi.org/10.1136/bmj.g1687.
2. Department of Health. The National Service Framework for Long Term Conditions. London: 2005.
3. Rumrill PD, Steffen JM, Kaleta DA, Holman CA. Job placement interventions for people with multiple sclerosis. Work 1996;6:167–75.
4. LaRocca NG, Kalb R, Gregg K. A program to facilitate retention of employment among persons with multiple sclerosis. vol. 7. 1996.
5. Sweetland J. Vocational Rehabilitation for People with Multiple Sclerosis. University College London, 2010.
6. Stimmel MB, Cohen JN, Schneider SJ, Portnoy JG, Seng EK, Foley FW. A neuropsychologically-based intervention with increased follow-up support for employed women with multiple sclerosis: a pilot randomized controlled trial. Clin Rehabil 2020;34:1292–302. https://doi.org/10.1177/0269215520940353.
7. Rumrill PD, Koch LC, Wohlford S. Job retention strategies for individuals with multiple sclerosis. J Vocat Rehabil 2013;39:127–35. https://doi.org/10.3233/JVR-130650.
8. Rumrill PD, Roessler R, Cook BG. Improving career re-entry outcomes for people with multiple sclerosis: A comparison of two approaches. J Vocat Rehabil 1998;10:241–52. https://doi.org/10.1016/S1052-2263(98)00020-8.
9. Rumrill PD, Fraser RT, Johnson KL. Employment and workplace accommodation outcomes among participants in a vocational consultation service for people with multiple sclerosis. J Vocat Rehabil 2013;39:85–90. https://doi.org/10.3233/JVR-130646.
10. Fraser RT, Johnson EK, Clemmons DC, Getter A, Johnson KL, Gibbons L. Vocational rehabilitation in multiple sclerosis (MS): A profile of clients seeking services. Work 2003;21:69–76.
11. Fraser RT, Clemmons D, Gibbons L, Koepnick D. Predictors of vocational stability in multiple sclerosis. J Vocat Rehabil 2009;31:129–35. https://doi.org/10.3233/JVR-2009-481.
12. Dorstyn D, Roberts R, Murphy G, Kneebone I, Craig A, Migliorini C. Online Resource to Promote Vocational Interests Among Job Seekers With Multiple Sclerosis: A Randomized Controlled Trial in Australia. Arch Phys Med Rehabil 2018;99:272–80.
13. Dorstyn D, Roberts R, Murphy G, Kneebone I, Migliorini C, Craig A, et al. Piloting an email-based resource package for job seekers with multiple sclerosis. Disabil Rehabil 2017;39:867–73.
14. Chiu C, Tansey T, Chan F, Strauser D, Frain M, Arora S. Effect of Rehabilitation Technology Services on Vocational Rehabilitation Outcomes of Individuals With Multiple Sclerosis. Rehabilitation Research, Policy, and Education 2015;29:183–92. https://doi.org/10.1891/2168-6653.29.2.183.
15. LaRocca NG, Hall HL. Multiple sclerosis program: a model for neuropsychiatric disorders. New Dir Ment Health Serv 1990;45:49–64. https://doi.org/10.1002/yd.23319904507. 5
16. Fraser RT, Clemmons DC, Bennet F. Multiple Sclerosis: Psychological and Vocational Interventions. 2002.

**Supplementary Material 3: TIDieR Checklist**

Table S 2 TIDieR Checklist MSVR.

| Criteria | Description |
| --- | --- |
| Brief Name | MSVR- Multiple Sclerosis Vocational Rehabilitation |
| Why | **The problem:**   - The average age of diagnosis of multiple sclerosis is between 20-40 years of age. These are the prime working years of an adult^1,2^. - After 10 years with the condition, less than 50% of people with multiple sclerosis remain at work^3^. - There is inconclusive evidence of the effectiveness of vocational rehabilitation to support people with multiple sclerosis to remain at work^4^.   **Theory:**   - Biopsychosocial approach^5^. - Vocational rehabilitation recommendations for people with long-term neurological conditions^6-8^. - Work disability prevention (The Work Disability Paradigm) ^9^ - United Kingdom Equality Act 2010^10^.   **Intervention goals:**   - To support the person with multiple sclerosis at work for as long as they wish. - To reduce work instability. - Improve the knowledge about multiple sclerosis and how to self-manage symptoms for the person with multiple sclerosis and their employers (or co-workers) |
| What materials? | - Exploratory interview from Employment and Multiple Sclerosis: A guide to vocational exploration for Occupational Therapists - List of services to refer the person with multiple sclerosis. - Informational and educational resources from organisations such as UK Multiple Sclerosis Society, National Multiple Sclerosis Society, Citizens Advice, Disability Rights UK, Advisory, Conciliation and Arbitration Service, UK Government, and scientific publications. - Summary letter after each appointment. - End of intervention package with top tips. |
| What procedures | - The first step involves completing the initial interview. The aim is to understand demographic and professional information, multiple sclerosis, and work characteristics, discuss what is important for the person with multiple sclerosis at work, and identify barriers to job retention. This session should finish setting at least 3 SMART goals (specific, measurable, achievable, realistic/relevant and time)^11^. - People with multiple sclerosis can receive between 1 to 10 hours of individually tailored support over three months distributed according to their needs and preference. - At the end of the intervention, the person with multiple sclerosis will discuss progress made, and evaluate goals and future steps. - If a person with multiple sclerosis agrees to employer involvement, the employer will be contacted. Employers will complete an initial interview (up to 1 hour) and receive up to 3 hours of support over three months. - Not all people with multiple sclerosis will want to involve their employer in the intervention. Employers will only be contacted with the consent of the employee with multiple sclerosis.   **Intervention components:** The intervention will be tailored to the needs and characteristics of the person with multiple sclerosis and their job. The person with multiple sclerosis can select from a menu of intervention components according to their needs:   - Employer engagement. - Education about multiple sclerosis. - Education about legal rights. - Support with disclosure. - Fatigue management. - Cognition in multiple sclerosis. - Signposting to local and national resources. - Advice about reasonable adjustments. - MS and emotions (e.g., anxiety, stress). - Long-term career planning. - Engagement/Collaboration with other professionals. |
| Who provided? | The background and experience of the vocational rehabilitation therapist may vary according to the complexity of the intervention.  Vocational rehabilitation services are generally provided by Occupational Therapists (approximately 77%)^12^, but psychologists are sometimes also involved when the interventions are offered within a health service^12^. Doogan & Playford (2014), discussed how the needs of people with MS can be addressed in a stepped care model where basic needs can be addressed with information, and higher complex needs can be addressed by “specialist’s groups”. Occupational Therapists with experience working with people with multiple sclerosis would constitute part of these specialist groups. Thus, an assistant psychologist with experience working with people with multiple sclerosis, knowledge of disability discrimination law, the ability to identify compensatory strategies to manage common multiple sclerosis symptoms at work, and an in-depth understanding of multiple sclerosis can deliver the intervention to address problems included in the Level 1 (information and signposting) and 2 (self-management at work) described by Doogan & Playford (2014).  Level 3 (specialist vocational rehabilitation) would need the input of an Occupational Therapist with extensive experience delivering vocational rehabilitation for people with multiple sclerosis and other health conditions. Thus, those assistant psychologists who lack expertise in any of these areas should be mentored by an Occupational Therapist to facilitate the delivery of the intervention.  The Occupational Therapist and assistant psychologist could collaborate to address complex issues that require further expertise such as discrimination at work, long-term sick leave, employment tribunals, etc. |
| How/ Where | The interview and intervention sessions can be conducted *face-to-face* and/or via *telephone* or *videoconference* according to the preference of the person with multiple sclerosis. Sessions will be conducted one-to-one to maximise intervention time. Sessions will be booked as frequently as required according to need.  The assistant psychologist and person with multiple sclerosis /employers will select the session topic before the meeting, and a summary with actions will be complied by the assistant psychologist and sent electronically after each session.  There will be frequent communication (at least once a week) between the assistant psychologist and person with multiple sclerosis /employers to address new topics as they appear. |
| When and how much? | Person with multiple sclerosis:   - Initial interview (1 hour) - Up to 10 hours of vocational rehabilitation over three months. Not all people with multiple sclerosis will require 10 hours, as this will depend on the complexity of their needs.   Employer:   - Initial interview (1 hour) - Up to 3 hours of support distributed over three months. Not all employers will require all the hours of support, as this will depend on their needs. |
| Tailoring | - Delivery: Each person can select the date and delivery mode of the sessions. - Content: The content of the intervention will be tailored from a menu of intervention components. - Employer involvement: Not all people with multiple sclerosis will agree to involve their employer and this only optional for the intervention. - Length of sessions: It is estimated that the sessions will last between 30-60 minutes according to the topic and the relevance of the topic for the person with multiple sclerosis. - The intervention will be tailored to increase its acceptability and facilitate its incorporation into their schedules. |
| How well? | A proforma for each intervention session will be completed to record the length and content of the session. Information about the number of people with multiple sclerosis involving their employers in the intervention will be recorded. |

**References**

1. Rao SM, Leo GJ, Bernardin L, Unverzagt F. Cognitive dysfunction in multiple sclerosis. I. Frequency, patterns, and prediction. Neurology 1991;41:685–91. https://doi.org/10.1212/WNL.41.5.685.
2. Fraser RT, Clemmons DC, Bennet F. Multiple Sclerosis: Psychological and Vocational Interventions. 2002.
3. Cardone A. Having the conversation about work with people with multiple sclerosis: a guide for healthcare professionals. Neurodegener Dis Manag 2017;7:41–4. https://doi.org/10.2217/nmt-2017-0041.
4. Khan F, Ng L, Turner-Stokes L. Effectiveness of vocational rehabilitation intervention on the return to work and employment of persons with multiple sclerosis. Cochrane Database of Systematic Reviews 2009. https://doi.org/10.1002/14651858.CD007256.pub2.
5. Wade DT, Halligan PW. The biopsychosocial model of illness: A model whose time has come. Clin Rehabil 2017;31:995–1004. https://doi.org/10.1177/0269215517709890.
6. BSRM. BSRM Standards for Rehabilitation Services Mapped on to the National Service Framework for Long-Term Conditions. London: 2009.
7. BSRM. Vocational asessment and rehabilitation for people with long-term neurological conditions: recommendations for best practice. 2010.
8. Hayward K, Mateen BA, Playford ED, Eva G. Developing vocational rehabilitation services for people with long-term neurological conditions: Identifying facilitators and barriers to service provision. British Journal of Occupational Therapy 2019;82:337–47. https://doi.org/10.1177/0308022619830294.
9. Loisel P. Developing a new paradigm: Work disability prevention. ICOH Special Issue 2009:1–5.
10. Equality Act. Equality Act 2010. The Equality Act 2010:251.4
11. Bovend’Eerdt TJ, Botell RE, Wade DT. Writing SMART rehabilitation goals and achieving goal attainment scaling: a practical guide. Clin Rehabil 2009;23:352–61. https://doi.org/10.1177/0269215508101741.
12. Playford ED, Radford K, Burton C, Gibson A, Jellie B, Sweetland J, et al. Mapping Vocational Rehabilitation Services for people with Long term neurological conditions: Summary report. 2011.
